# Chronic Disease Self-Management Program: Insights from the Eye of the Storm

**DOI:** 10.3389/fpubh.2014.00253

**Published:** 2015-04-27

**Authors:** Kate Lorig

**Affiliations:** ^1^Patient Education Research Center, School of Medicine, Stanford University, Stanford, CA, USA

**Keywords:** self-management, patient education, CDSMP, chronic disease, translational research

In the early 1990s, the Stanford Patient Education Research Center developed the Chronic Disease Self-Management Program (CDSMP) to test the hypothesis that people with comorbid conditions could benefit when placed in a common intervention. At that time, the existing paradigm consisted of having patients attend only disease-specific education programs. In 2013 alone, 50–100,000 people in 36 countries attended the CDSMP. How did this happen? We do not know the answer, but have some ideas. The following is a brief history and some key insights.

## Development

In 1990, to determine patient-perceived problems, we held 11 focus groups with people with chronic conditions. Participants talked predominately about symptoms, and thus the program was built around breaking the symptom cycle and tools that participants could use to accomplish this. By basing a program on end-user problems, we assured their interest. Insight: one cannot underestimate the importance of having happy and excited end-users. This can only be accomplished by meeting user needs.

We developed the CDSMP for translation into practice. It is taught by peers. Every minute was and continues to be scripted for both content and process. Insight: the design process accounted for many of the things that enabled the CDSMP to be a success. Translation cannot occur without a set protocol that can be followed by others.

The CDSMP was based on self-efficacy (SE) theory. While many interventions are informed by theory, the CDSMP systematically incorporated SE theory. SE theory states that one’s confidence in achieving a desired behavior predicts their level of success. SE can be enhanced through skills mastery, modeling, reinterpretation, and social persuasion (1). All of these are used throughout the program. For example, participants made action plans (skills mastery) and shared with other participants their confidence in achieving their plan each week. If a participant’s confidence was low, then the leaders and other participants helped them problem-solve (2). Insight: theories are useful – but only if theories are translated into programmatic elements.

The original randomized trial had four outcome categories that were of interest to different communities (3). Behaviors such as exercise were of interest to the behavioral science community as was SE. Symptom-based outcomes (pain, depression, fatigue) were of interest to patients and health-care providers, while changes in utilization, such as days in hospital and emergency department visits were of interest to health service researchers, government, and others who pay for health care. Insight: choose outcomes that are of interest to communities and policy makers you hope will use and adopt your program.

## Early Replication

At the end of the original randomized trial, there were improvements in all four categories. Hospitalization was reduced by 8 days. Based on these data, Kaiser Permanente, one of the original study partners, decided to trial the program nationally in 1998. This longitudinal study had similar outcomes to the original trial (4). Insight: having a respected partner who is also an early adopter gives translation a head start.

At about this same time, having read our original article, others from around the country began to call inquiring about the program. Insight: publish as soon as practical using language understandable outside the scientific community. Publications should be aimed at scientific, practice, and policy communities.

Based on this interest, we started offering one or two yearly trainings in 1999. Our aim was to give each organization the capacity to train its own leaders and to grow its own program. As developers, we saw our role as offering training and technical assistance. What began with 20–40 trainers per year has grown to 400 or more new trainers per year. Each pair of new trainers has the capacity to offer programs and train local leaders. Insight: building organizational capacity is an important translation element. To do this, one must devote resources to training and supporting others.

## Legal Stuff and Agility

By the early twenty-first century, requests for training were rapid. There was a need to put more structure around the translation process. There has never been a business plan. Rather, the business of translation was and continues to respond to changing needs. Early on, Stanford administration showed little interest in our activities. As we involved more organizations, the University became concerned about liability. To mitigate this issue, we worked with the Stanford Office of Technology Licensing to establish policies. Insights: program developers need to worry about liability and licensure issues.

There were five potentially competing interests, the legal interests of the University, the need to keep the workshops affordable for adopting agencies, the need to sustain a training technical-assistance (TA) infrastructure, the financial interests of the program developers, and the need to minimize bureaucracy. At this stage in translation, many program developers form their own companies or collaborate with an existing company. However, the developers were not interested in becoming entrepreneurs. We decided to continue working within the University. License price ($500) was set to allow an agency to offer 30 workshops over 3 years for approximately 300 participants. Insight: in translating products to widespread use, there are many competing interests. It is best to acknowledge these and work at a fair compromise early.

Between 2000 and 2010, both the licensing and training policies adapted to changing times and became more codified. With the help of the Office for Technology Licensing, we created and continue to create different types of licenses. See http://patienteducation.stanford.edu/licensing for current license policy and http://patienteducation.stanford.edu/training/trnpolicies.html for training policy. Insight: while personal preference and knowledge can run early translation efforts, true widespread translation requires “rules and regulations.”

## Policy

In 2003, the U.S. Administration on Aging, AoA (now a unit of the Administration for Community Living, ACL) in collaboration with CDC and other public and philanthropic organizations, funded 14 sites to embed evidence-based programs into community-based organizational networks. It was only after several of the applicants wanted to use the CDSMP that the head of the National Council on Aging TA Center for these grants called Stanford. Until this time, no one at Stanford knew anything about this initiative. Because of this collaboration, more than 3000 people had participated in evidence-based programs including the CDSMP (5). Insight: sometimes adoption on a national level comes from the grass roots up.

In 2006, the U.S. Department of Health and Human Services announced collaboration between AoA, NCOA, and the Atlantic Philanthropies to build CDSMP capacity across the United States. AoA awarded funding to 27 states. This funding mandated adoption of the CDSMP and encouraged the use of other evidence-based programs. These programs served approximately 50,000 people (6). Between 2005 and 2010, organizations not funded by AoA also began to offer the CDSMP. These included major health plans, a third-party insurer and local agencies. In 2010, as part of the *American Recovery and Reinvestment Act*, ARRA recovery funding, AoA in collaboration with CDC, provided grants to 45 states, Puerto Rico and the District of Columbia for disseminating the CDSMP. The goal of 50,000 completers (those who had attended four or more sessions out of six) was reached and surpassed. Insight: even in bad times good things can happen. Insight: when opportunity knocks it is important to have “shovel ready” projects.

As part of the AARA funding, the CDSMP was evaluated in a large study involving 22 organizationally and geographically diverse sites. The outcomes demonstrated that the program continued to meet the triple aims of health care, better care, better health, and lower costs (7). Following ARRA, more secure funding was achieved in the AoA (now ACL) budget. Authorizing legislation in the Older Americans Act has long included Title IIID for Disease Prevention and Health Promotion. Beginning in 2012, ACL required states to use these funds ($21,000,000) for evidence-based programs. Also in 2012, CDSMP became a small line item in the AoA (now ACL) budget financed through the 2012 Affordable Care Act Prevention and Public Health Fund. While the funding was much reduced from that received from AARA, 22 states received grants. Some states that had been funded under AARA were not refunded through these grants. However they continue to offer the CDSMP utilizing IIID and other monies coming from many sources. These include foundations, health care, and other local, state, and federal agencies. UniteHere, a union of mostly low paid service workers, recently completed its second year of offering the CDSMP, mostly in Spanish. They have reached several hundred workers in the Los Angeles area and are currently expanding the program to their members in many other cities. Insight: if a program meets local needs and is liked by both agencies and participants, there is life even when funding is reduced. Insight: if grant funds can build capacity and engagement, sometimes programs can be sustained through other sources.

## Current Challenges

In 2014, the CDSMP continues to gather momentum. It has multiple funders among U.S. federal agencies as well as U.S. foundations and health-care systems. As the program has grown, so have the challenges for its creators. (Please note that there are many other challenges for those offering the programs.)

### Encouraging and discouraging adaptations

There is constant pressure to adapt and modify the CDSMP. These requests usually come from people who have not seen or participated in the program and usually know “what is best for *my population*.” These requests range from wanting to change content to changing length or format. Insight: there is distrust of anything “Not Invented Here.”

We usually tell the requesters to try the program, and then ask the participants what they want to change. Requests for changes in format and length or large amounts of content cannot be met without rewriting the CDSMP and re-evaluating the new format with a new population. This has been done successfully a few times and has resulted in the pain self-management program and the hepatitis-c self-management program, among others (8, 9). Recently, we have encouraged groups wanting to make changes to ask permission for small, rapid-change cycle experiments and to report the finding. However, few have conducted such experiments. Insight: when given a process rather than permission for making change, there is little uptake.

## Fidelity

Evidence-based programs always have the challenge of standardization. Without standardization, the evidence base is lost. As the core of trainers has grown larger (over 1000 master trainers and many thousand leaders), maintaining quality programs is more difficult. The use of webinars, administrative and fidelity manuals, and email discussion groups helps with the centralization of key training and technical assistance (10, 11). Insight: fidelity is a delicate balance that constantly has to be re-evaluated and maintained.

It is unusual for a program creator to remain involved with widespread translation. There have been several challenges. First of these is moving among academic, training, technical assistance, promoting, and cheerleading roles. Insight: if you do not like juggling, do not join the circus.

The second is how to finance core translations activities such as training, technical assistance, and updating materials. Monies from federal agencies and foundations, for the most part, go for program delivery and are seldom earmarked for these core activities. This means that the core functions must become self-sustaining through charging for such activities as training, materials, and TA. Insight: the financing of core translation activities can help or hinder translation and must be planned and flexible.

## Summary

This is a personal 22-year retrospective look at insights gained as the CDSMP has moved from concept to translation. This retrospective look has been both surprising and humbling. I look forward to learning what comes next.

## Conflict of Interest Statement

The author receives royalties from Stanford University and Bull Publishing.

This paper is included in the Research Topic, “Evidence-Based Programming for Older Adults.” This Research Topic received partial funding from multiple government and private organizations/agencies; however, the views, findings, and conclusions in these articles are those of the authors and do not necessarily represent the official position of these organizations/agencies. All papers published in the Research Topic received peer review from members of the Frontiers in Public Health (Public Health Education and Promotion section) panel of Review Editors. Because this Research Topic represents work closely associated with a nationwide evidence-based movement in the US, many of the authors and/or Review Editors may have worked together previously in some fashion. Review Editors were purposively selected based on their expertise with evaluation and/or evidence-based programming for older adults. Review Editors were independent of named authors on any given article published in this volume.
