# Corrigendum: “Chronic Disease Self-Management Program: Insights from the Eye of the Storm”

**DOI:** 10.3389/fpubh.2015.00153

**Published:** 2015-06-01

**Authors:** Kate Lorig

**Affiliations:** ^1^Patient Education Research Center, School of Medicine, Stanford University, Stanford, CA, USA

**Keywords:** corrigendum, self-management, patient education, CDSMP, chronic disease, translational research

There is an error in the original manuscript under the Section “Early Replication”. The number of days of reduced hospitalization should be 0.8, not 8 as originally published.

## Conflict of Interest Statement

The author receives royalties from Stanford University and Bull Publishing.

This paper is included in the Research Topic, “Evidence-Based Programming for Older Adults.” This Research Topic received partial funding from multiple government and private organizations/agencies; however, the views, findings, and conclusions in these articles are those of the authors and do not necessarily represent the official position of these organizations/agencies. All papers published in the Research Topic received peer review from members of the Frontiers in Public Health (Public Health Education and Promotion section) panel of Review Editors. Because this Research Topic represents work closely associated with a nationwide evidence-based movement in the US, many of the authors and/or Review Editors may have worked together previously in some fashion. Review Editors were purposively selected based on their expertise with evaluation and/or evidence-based programming for older adults. Review Editors were independent of named authors on any given article published in this volume.

